# Supplementary material for: Heat adaptation of phage T7 under an extended genetic code
Source: Virus Evol. 2021 Dec 1;7(2):veab100. doi: 10.1093/ve/veab100 (PMC8923235; doi:10.1093/ve/veab100)
Supplement: veab100_Supp [file veab100_supp.zip › Sequencing_Analysis.zip!/Sequencing_Analysis/S3/evidence/JC_45.html]

BRESEQ :: Evidence


| New junction evidence | | | | | | | | | | | |
| --- | --- | --- | --- | --- | --- | --- | --- | --- | --- | --- | --- |
|  | | seq id | position | reads (cov) | reads (cov) | score | skew | freq | annotation | gene | product |
| \* | ? | NC\_001604.1 | 295 = | 2222 (1.290) | 18 (0.010) | 13/560 | 37.9 | 0.86% | intergenic (–/–) | *–/–* | –/– |
|  | ? | NC\_001604.1 | = 281 | 2083 (1.300) |  |  |  |  | intergenic (–/–) | *–/–* | –/– |
| Rejected: Coverage evenness skew score above cutoff. | | | | | | | | | | | |
| Rejected: Frequency below/above cutoff threshold. | | | | | | | | | | | |

Only 100 of 273 total aligned reads displayed.  

`TACTGCATGTTACTTCATGTTGTCAATACCTGTTTTTCGTGCGACTTATCAGGCTGTCTACTTATCCGGTTGACCCCGGTGTCGATTGGGATGGCTATTCGCCGTGTCCCTCTCGATGGCTGTAAGTATCCTATAGGTTAGACTTTAAGTCAATACTCTTTTTGATAAATTTTAAATTAATCTTTTAAGTCTCTTTAAGTTGTCTCTCCTTATAGTGAGTTGTATTAATAAAGACAATGATTAAAGGTTATCACTTTATGGCTAACAACAAGGCGTCTTTAGGTCTGGTCTTTATGTAG‑TGTCTTTAGGTCTGGTCTTTA‑‑‑‑‑‑‑‑‑‑‑‑‑‑‑‑‑‑‑‑‑‑‑‑‑‑‑‑‑‑‑‑‑‑‑‑‑‑‑‑‑‑‑‑‑‑‑‑‑‑‑‑‑‑‑‑‑‑‑‑‑‑‑‑‑‑‑‑‑‑‑‑‑‑‑‑‑‑‑‑‑‑‑‑‑‑‑‑‑‑‑‑‑‑‑‑‑‑‑‑‑‑‑‑‑‑‑‑‑‑‑‑‑‑‑‑‑‑‑‑‑‑‑‑‑‑‑‑‑‑‑‑‑‑‑‑‑‑‑‑‑‑‑‑‑‑‑‑‑‑‑‑‑‑‑‑‑‑‑‑‑‑‑‑‑‑‑‑‑‑‑‑‑‑‑‑‑‑‑‑‑‑‑‑‑‑‑‑‑‑‑‑‑‑‑‑‑‑‑‑‑‑‑‑‑‑‑‑‑‑‑‑‑‑‑‑‑‑‑‑‑‑‑‑‑‑‑‑‑‑‑‑‑‑‑‑‑‑‑‑‑‑‑‑‑‑‑‑‑‑‑‑‑‑‑‑‑‑‑‑‑‑‑‑‑‑‑‑‑‑‑‑‑‑‑‑‑‑‑‑‑‑‑‑‑‑‑‑‑‑‑‑‑‑  <  NC_001604.1/614‑295`  
`‑‑‑‑‑‑‑‑‑‑‑‑‑‑‑‑‑‑‑‑‑‑‑‑‑‑‑‑‑‑‑‑‑‑‑‑‑‑‑‑‑‑‑‑‑‑‑‑‑‑‑‑‑‑‑‑‑‑‑‑‑‑‑‑‑‑‑‑‑‑‑‑‑‑‑‑‑‑‑‑‑‑‑‑‑‑‑‑‑‑‑‑‑‑‑‑‑‑‑‑‑‑‑‑‑‑‑‑‑‑‑‑‑‑‑‑‑‑‑‑‑‑‑‑‑‑‑‑‑‑‑‑‑‑‑‑‑‑‑‑‑‑‑‑‑‑‑‑‑‑‑‑‑‑‑‑‑‑‑‑‑‑‑‑‑‑‑‑‑‑‑‑‑‑‑‑‑‑‑‑‑‑‑‑‑‑‑‑‑‑‑‑‑‑‑‑‑‑‑‑‑‑‑‑‑‑‑‑‑‑‑‑‑‑‑‑‑‑‑‑‑‑‑‑‑‑‑‑‑‑‑‑‑‑‑‑‑‑‑‑‑‑‑‑‑‑‑‑‑‑‑‑‑‑‑‑‑‑‑‑‑‑‑‑‑‑‑‑‑‑‑‑‑‑‑‑‑‑‑‑‑‑‑‑‑‑‑‑‑‑‑‑‑‑‑‑‑‑‑‑‑gtctttaggtctggtctttaTGTTTAAACTTTAAGATAGGCGTTGACTTGATGGGTCTTTAGGTGTAGGCTTTAGGTGTTGGCTTTAGGATGGACGTTAGGAGGTGACTTTAGGAGGATACTTTAGGAGACTGTAACAGATAGGGACACAGAGAGACACTCAAGGTAACACCCAAAGACAAACCCAAGGGTCATCCCCAACCCTTAGGGAACCCTCAAGGTCAACCGAAGGTTGACTTTAGGTGATTGGCTGGGCTTTAGGTACCCCCTATGGGGGAACTTTAGGTCCGTACACTGTGAGA‑‑‑‑‑‑‑‑‑‑‑‑‑  <  NC_001604.1/281‑1`  

                                                                                                                                                                                                                                                                                                                                                                                                                                                                                                                                                                                                                                         
`tataagagacagCTTCATGTTGTCAATACCTGTTTTTCGTGCGACTTATCAGGCTGTCTACTTATCCGGTTGACCCCGGTGTCGATTGGGATGGCTATTCGCCGTGTCCCTCTCGATGGCTGTAAGTATCCTATAGGTTAGACTTTAAGTCAATACTCTTTTTGATAAATTTTAAATTAATCTTTTAAGTCTCTTTAAGTTGTCTCTCCTTATAGTGAGTTGTATTAATAAAGACAATGATTAAAGGTTATCACTTTATGGCTAACAACAAGGCGTCTTTAGGTCTGGTCTTTATGTAG‑TG                                                                                                                                                                                                                                                                                                                           <  1:128225/289‑1`  
 `ACTGCTTGTTACTTCATGTTGTCAATACCTGTTTTTCGTGCGACTTATCAGGCTGTCTACGTATCCGGTTGACCCCGGTGTCGATTGGGATGGCTATTCGCCGTGTCCCTCTCGATGGATGTAAGTATCCTATAGGTTAGACTTTAAGTCAATACTCTTTTTGATAAATTTTAAATTAATCTTTTAAGTCTCTTTAAGTTGTCTCTCCTTATAGTGAGTTGTATTAATAAAGACAATGATTAAAGGTTATCACTTTATGGCTAACAACAAGGCGTCTTTAGGTCTGGTCTTTATGTAG‑TGT                                                                                                                                                                                                                                                                                                                          <  1:310450/301‑1`  
 `ACTGCATGTTACTTCATGTTGTCAATACCTGTTTTTCGTGCGACTTATCAGGCTGTCTACTTATCCGGTTGACCCCGGTGTCGATTGGGATGGCTATTCGCCGTGTCCCTCTCGATGGCTGTAAGTATCCTATAGGTTAGACTTTAAGTCAATACTCTTTTTGATAAATTTTAAATTAATCTTTTAAGTCTCTTTAAGTTGTCTCTCCTTATAGTGAGTTGTATTAATAAAGACAATGATTAAAGGTTATCACTTTATGGCTAACAACAAGGCGTCTTTAGGTCTGGTCTTTATGTAG‑TGT                                                                                                                                                                                                                                                                                                                          >  1:230950/1‑301`  
 `ACTGCATGTTACTTCATGTTGTCAATACCTGTTTTTCGTGCGACTTATCAGGCTGTCTACTTATCCGGTTGACCCCGGTGTCGATTGGGATGGCTATTCGCCGTGTCCCTCTCGATGGCTGTAAGTATCCTATAGGTTAGACTTTAAGTCAATACTCTTTTTGATAAATTTTAAATTAATCTTTTAAGTCTCTTTAAGTTGTCTCTCCTTATAGTGAGTTGTATTAATAAAGACAATGATTAAAGGTTATCACTTTATGGCTAACAACAAGGCGTCTTTAGGTCTGGTCTTTATGTAG‑TGT                                                                                                                                                                                                                                                                                                                          >  1:11339/1‑301`  
 `ACTGCATGTTACTTCATGTTGTCAATACCTGTTTTTCGTGCGACTTATCAGGCTGTCTACTTATCCGGTTGACCCCGGTGTCGATTGGGATGGCTATTCGCCGTGTCCCTCTCGATGGCTGTAAGTATCCTATAGGTTAGACTTTAAGTCAATACTCTTTTTGATAAATTTTAAATTAATCTTTTAAGTCTCTTTAAGTTGTCTCTCCTTATAGTGAGTTGTATTAATAAAGACAATGATTAAAGGTTATCACTTTATGGCTAACAACAAGGCGTCTTTAGGTCTGGTCTTTATGTAG‑TGT                                                                                                                                                                                                                                                                                                                          <  1:246246/301‑1`  
 `ACTGCATGTTACTTCATGTTGTCAATACCTGTTTTTCGTGCGACTTATCAGGCTGTCTACTTATCCGGTTGACCCCGGTGTCGATTGGGATGGCTATTCGCCGTGTCCCTCGCGATGGCTGTAAGTATCCTATAGGTTAGACTTTAAGTCAATACTCTTTTTGATAAATTTTAAATTAATCTTTTAAGTCTCTTTAAGTTGTCTCTCCTTATAGTGAGTTGTATTAATAAAGACAATGATTAAAGGTTATCACTTTATGGCTAACAACAAGGCGTCTTTAGGTCTGGTCTTTATGTAG‑TGT                                                                                                                                                                                                                                                                                                                          <  1:139123/301‑1`  
    `GCATGTTACTTCATGTTGTCAATACCTGTTTTTCGTGCGACTTATCAGGCTGTCTACTTATCCGGTTGACCCCGGTGTCGATTGGGATGGCTATTCGCCGTGTCCCTCTCGATGGCTGTAGGTATCCTATAGGTTAGACTTTAAGTCAATACTCTTTTTGATAAATTTTAAATTAATCTTTTAAGTCTCTTTAAGTTTTCTCTCCTTATAGTGAGTTTTATTAATAAAGACAATGATTAAAGGTTATCACTTTATGGCTAACAACAAGGCGTCTTTAGGTCTGGTCTTTATGTAG‑TGTCTT                                                                                                                                                                                                                                                                                                                       >  1:271780/1‑301`  
    `GCATGTTACTTCATGTTGTCAATACCTGTTTTTCGTGCGACTTATCAGGCTGTCTACTTATCCGGTTGACCCCGGTGTCGATTGGGATGGCTATTCGCCGTGTCCCTCTCGATGGCTGTAAGTATCCTATAGGTTAGACTTTAAGTCAATCCTCTTTTTGATAAATTTTAAATTAATCTTTTAAGTCTCTTTTCGTTGTCTCTCCTTATAGTGCGTTGTCTTAATAAAGACAATGATTAAAGGTTATCACTTTATGGCTCACAACCAGGCGTCTTTAGGTCTGGTCTTTATGTAG‑TGTCTT                                                                                                                                                                                                                                                                                                                       >  1:281444/1‑301`  
    `GCATGTTACTTCATGTTGTCAATACCTGTTTTTCGTGCGACTTATCAGGCTGTCTACTTATCCGGTTGACCCCGGTGTCGATTGGGATGGCTATTCGCCGTGTCCCTCTCGATGGCTGTAAGTATCCTATAGGTTAGACTTTAAGTCAATACTCTTTTTGATAAATTTTAAATTAATCTTTTAAGTCTCTTTAAGTTGTCTCTCCTTATAGTGAGTTGTATTAATAAAGACAATGATTAAAGGTTATCACTTTATGGCTAACAACAAGGCGTCTTTAGGTCTGGTCTTTATGTAG‑TGTCTT                                                                                                                                                                                                                                                                                                                       >  1:295137/1‑301`  
    `GCATGTTACTTCATGTTGTCAATACCTGTTTTTCGTGCGACTTATCAGGCTGTCTACTTATCCGGTTGACCCCGGTGTCGATTGGGATGGCTATTCGCCGTGTCCCTCTCGATGGCTGTAAGTATCCTATAGGTTAGACTTTAAGTCAATACTCTTTTTGATAAATTTTAAATTAATCTTTTAAGTCTCTTTAAGTTGTCTCTCCTTATAGTGAGTTGTATTAATAAAGACAATGATTAAAGGTTATCACTTTATGGCTAACAACAAGGCGTCTTCAGGTCTGGTCTTTATGTAG‑TGTCTT                                                                                                                                                                                                                                                                                                                       >  1:252779/1‑301`  
    `GCATGTTACTTCATGTTGTCAATACCTGTTTTTCGTGCGACTTATCAGGCTGTCTAC‑TATCCGGTTTACCCCGGTGTCGATTGGGATGGCTATTCGCCGTGTCCCTCTCGATGGCTGTAAGTATTCTATAGGTTAGACTTTAAGTCAATACTCTTTTTGATAAATTTTACATTAATCTTTTAAGTCTCTTTAAGTTGTCTCTCCTTATCGTCAGTTGTATTAATAAAGACAATGATTAAAGGTTATTACTTTATGGCTAACAACAAGTCGTCTTTAGGTCTGGTCTTTATGTAG‑TGTCTTT                                                                                                                                                                                                                                                                                                                      >  1:161129/1‑301`  
     `CATGTTACTTCATTTTGTCAATACCTGTTTTTCGTGCGACTTATCAGTCTGTCTACTTATTCGGTTGCCCCCGGTGTCGATTGGGATGGCTATTCGCCGTGTCCCTCTCGATGGCTGTAAGTATCCTATAGGTTAGACTTTAAGTCAATACTCTTTTTGATAAATTTTAAATTAATCTTTTAAGTCTCTTTAAGTTGTCTCTCCTTATAGTGAGTTGTATTAATAAAGACAATGATTAAAGGTTATCACTTTATGGCTAACAACAAGGCGTCTTTAGGTCTGGTCTTTATGTAG‑TGTCTTT                                                                                                                                                                                                                                                                                                                      <  1:23578/301‑1`  
      `ATGTTACTTCATGTTGTCAATACCTGTTTTTCGTGCGACTTATCAGGCTGTCTACTTATCCGGTTGACCCCGGTGTCGATTGGGATGGCTATTCGCCGTGTCCCTCTCGATGGCTGTAAGTATCCTATAGGTTAGACTTTAAGTCAATACTCTTTTTGATAAATTTTAAATTAATCTTTTAAGTCTCTTTAAGTTGTCTCTCCTTATAGTGAGTTGTATTAATAAAGACAATGATTAAAGGTTATCACTTTATGGCTAACAACAAGGCGTCTTTAGGTCTGGTCTTTATGTAGCTGTCTct                                                                                                                                                                                                                                                                                                                      >  1:25013/1‑299`  
      `ATGTTACTTCATGTTGTCAATACCTGTTTTTCGTGCGACTTATCAGGCTGTCTACTTATCCGGTTGACCCCGGTGTCGATTGGGATGGCTATTCGCCGTGTCCCTCTCGATGGCTGTAAGTATCCTATAGGTTAGACTTTAAGTCAATACTCTTTTTGATAAATTTTAAATTAATCTTTTAAGTCTCTTTAAGTTGTCTCTCCTTATAGTGAGTTGTATTAATAAAGACAATGATTAAAGGTTATCACTTTATGGCTAACAACAAGGCGTCTTTAGGTCTGGTCTTTATGTAG‑TGTCTTTt                                                                                                                                                                                                                                                                                                                     >  1:12713/1‑300`  
      `ATGTTACTTCATGTTGTCAATACCTGTTTTTCGTGCGACTTATCAGGCTGTCTACTTATCCGGTTGACCCCGGTGTCGATTGGGATGGCTATTCGCCGTGTCCCTCTCGATGGCTGTAAGTATCCTATAGGTTAGACTTTAAGTCAATACTCTTTTTGATAAATTTTAAATTAATCTTTTAAGTCTCTTTAAGTTGTCTCTCCTTATAGTGAGTTGTATTAATAAAGACAATGATTAAAGGTTATCACTTTATGGCTAACAACAAGGCGTCTTTAGGGCTGGTCTTTATGTAG‑TGACTTTA                                                                                                                                                                                                                                                                                                                     >  1:83707/1‑301`  
        `ttataagagacagGTCAATACCTGTTTTTCGTGCGACTTATCAGGCTGTCTACTTATCCGGTTGACCCCGGTGTCGATTGGGATGGCTATTCGCCGTGTCCCTCTCGATGGCTGTAAGTATCCTATAGGTTAGACTTTAAGTCAATACTCTTTTTGATAAATTTTAAATTAATCTTTTAAGTCTCTTTAAGTTGTCTCTCCTTATAGTGAGTTGTATTAATAAAGACAATGATTAAAGGTTATCACTTTATGGCTAACAACAAGGCGTCTTTAGGTCTGGTCTTTATGTAG‑TGTCTTTAGG                                                                                                                                                                                                                                                                                                                   <  1:239498/288‑1`  
        `ttataagagacagGTCAATACCTGTTTTGCGTGCGACTTATCAGGCTGTCTACTTATCCGGTTGACCCCGGTGTCGATTGGGATGGCTATTCGCCGTGTCCCTCTCGATGGCTGTAAGTATCCTATAGGTTAGACTTTAAGTCAATACTCTTTTTGATAAATTTTAAATTAATCTTTTAAGTCTCTTTAAGTTGTCTCTCCTTATAGTGAGTTGTATTAATAAAGACAATGATTAAAGGTTATCACTTTATGGCTAACAACAAGGCGTCTTTAGGTCTGGTCTTTATGTAG‑TGTCTTTAGG                                                                                                                                                                                                                                                                                                                   <  1:37301/288‑1`  
        `tTTACTTCATGTTGTCAATACCTGTTTTTCGTGCGACTTATCAGGCTGTCTACTTATCCGGTTGACCCCGGTGTCGATTGGGATGGCTATTCGCCGTGTCCCTCTCGATGGCTGTAAGTATCCTATAGGTTAGACTTTAAGTCAATACTCTTTTTGATAAATTTTAAATTAATCTTTTAAGTCTCTTTAAGTTGTCTCTCCTTATAGTGAGTTGTATTAATAAAGACAATGATTAAAGGTTATCACTTTATGGCTAACAACAAGGCGTCTTTAGGTCTGGTCTTTATGTAG‑TGTCTTTAGG                                                                                                                                                                                                                                                                                                                   <  1:2028/300‑1`  
        `tTTACTTCATGTTGTCAATACCTGTTTTTCGTGCGACTTATCAGGCTGTCTACTTATCCGGTTGACCCCGGTGTCGATTGGGATGGCTATTCGCCGTGTCCCTCTCGATGGCTGTAAGTATCCTATAGGTTAGACTTTAAGTCAATACTCTTTTTGATAAATTTTAAATTAATCTTTTAAGTCTCTTTAAGTTGTCTCTCCTTATAGTGAGTTGTATTAATAAAGACAATGATTAAAGGTTATCACTTTATGGCTAACAACAAGGCGTCTTTAGGTCTGGTCTTTATGTAG‑TGTCTTTAGG                                                                                                                                                                                                                                                                                                                   <  1:338064/300‑1`  
        `cagACTTCATGTTTTCAATACCTGTTTTTCGTGCGACTTATCAGGCTGTCTACTTATCCGGTTGACCCCGGTGTCGATTGGGATGGCTATTCGCCGTGTCCCTCTCGATGGCTGTAAGTATCCTATAGGTTAGACTTAAAGTCAATACTCTTTTTGATAAATTTTAAATTAATCTTTTAAGTCTCTTTAAGTTGTCTCTCCTTATAGTGAGTTGTATTAATAAAGACAATGATTAAAGGTTATCACTTTATGACTAACAACAAGGCGTCTTTAGGTCTGGTCTTTATGTAG‑TGTCTTTAGG                                                                                                                                                                                                                                                                                                                   <  1:318559/298‑1`  
        `cagACTTCATGTTGTCAATACCTGTTTTTCGTGCGACTTATCAGGCTGTCTACTTATCCGGTTGACCCCGGTGTCGATTGGGATGGCTATTCGCCGTGTCCCTCTCGATGGCTGTAAGTATCCTATAGGTTAGACTTTAAGTCAATACTCTTTTTGATAAATTTTAAATTAATCTTTTAAGTCTCTTTAAGTTGTCTCTCCTTATAGTGAGTTGTATTAATAAAGACAATGATTAAAGGTTATCACTTTATGGCTAACAACAAGGCGTCTTTAGGTCTGGTCTTTATGTAG‑TGTCTTTAGG                                                                                                                                                                                                                                                                                                                   <  1:69987/298‑1`  
        `GTTACTTCATGTTGTCAATAGCTGTTTTTCGTGCGACTTATCAGGCTGTCTACTTATCCGGTTGACCCCGGTGTCGATTGGGATGGCTATTCGCCGTGTCCCTCTCGATGGCTGTAAGTATCCTATAGGTTAGACTTTAAGTCAATACTCTTTTTGATAAATTTTAAATTAATCTTTTAAGTCTCTTTAAGTTGTCTCTCCTTATAGTGAGTTGTATTAATAAAGACAATGATTAAAGGTTATCACTTTATGGCTAACAACAAGGCGTCTTTAGGTCTGGTCTTTATGTAG‑TGTCTTTAGG                                                                                                                                                                                                                                                                                                                   <  1:76699/301‑1`  
        `GTTACTTCATGTTGTCAATACCTGTTTTTCGGGCGACTTATCAGGCTGTCTACTTATCCGGTTGACCCCGGTGTCGATTGGGATGGCTATTCGCCGTGTCCCTATCGATGGCTGTAAGTATCCTATAGGTTAGACTTTAAGTCAATACTCTTTTTGATAAATTTTAAATTAATCTTTTAAGTCTCTTCAAGTTGTCTCTCCTTATAGTGAGTTGTATTAATAAAGACAATGATTAAAGGTTATCACTTTATGGCTAACAACAAGGCGTCTTTAGGTCTGGTCTTTATGTAG‑TGTCTTTAGG                                                                                                                                                                                                                                                                                                                   <  1:66167/301‑1`  
         `TTACTTCATGTTGTCAATACCTGTTTTTCGTGCGACTTATCAGGCTGTCTACTTATCCGGTTGACCCCGGTGTCGATTGTGATGGCTATTCGCCGTGTCCCCCTCGATGGCTGTAATTATCCTATAGGTTAGACTTTAACTCAATACTCTTTTTCCTAAATTTTAAATTAATCTTTTAAGTCTCTTTAAGTTGTCTCTCCTTCTAGTGAGTTGTCTTAATAAAGACAATGATTAACGGTTATGACTTTATGGCTAACACCAAGTCGTCTTTATGTCTGGTTTTTTTGTAT‑TATATTTAGGT                                                                                                                                                                                                                                                                                                                  >  1:184568/1‑301`  
         `TTACTTCATGTTGTCAATACCTGTTTTTCGTGCGACTTATCAGGCTGTCTACTTATCCGGTTGACCCCGGTGTCGATTGGGATGGCTATTCGCCGTGTCCCTCTCGATGGCTGTAAGTATCCTATAGGTTAGACTTTAAGTCAATACTCTTTTTGATAAATTTTAAATTAATCTTTTAAGTCTCTTTAAGTTGTCTCTCCTTATAGTGAGTTGTATTAATAAAGACAATGATTAAAGGTTATCACTTTATGGCTAACAACAAGGCGTCTTTAGATCTGGTCTTTATGTAG‑TGTCTTTAGGT                                                                                                                                                                                                                                                                                                                  >  1:109380/1‑301`  
         `TTACTTCATGTTGTCAATACCTGTTGTTCGTGCGACTGATCAGGCTGTCTACTTATCCGGTTGACCCCGGTGTCGATTGGGATGGCTATTCGCCGTGTCCCTCTCGATGGCTGTAAGTATCCTATAGGTTAGACTTTAAGTCAATACTCTTTTTGTTAAATTTTAAATTAATCTTTTAAGTCTCTTTAAGTTGTCTCTCCTTATAGTGAGTTGTATTAATAAAGACAATGATTAAAGGTTATCACTTTATGGCTAACAACAAGGCGTCTTTAGGTCTGGTCTTTATGTAG‑TGTCTTTAGGT                                                                                                                                                                                                                                                                                                                  <  1:330209/301‑1`  
         `TTACTTCATGTTGTCAATACCTG‑TTTTCGTGCGACTTATCAGGCTGTCTACTTATCCGGTTGACCCCGGTGTCGATTGGGATGGCTATTCGCCGTGTCCCTCTCGATGGCTGTAAGTATCCTATAGGTTAGACTTTAAGTCAATACTCTTTTTGATAAATTTTAAATTAATCTTTTAAGTCTCTTTAAGTTGTCTCTCCTTATAGTGAGTTGTATTAATAAAGACAATGATTAAAGGTTATCACTTTATGGCTAACAACAAGGCGTCTTTAGGTCTGGTCTTTATGTAG‑TGTCTTTAGGTC                                                                                                                                                                                                                                                                                                                 <  1:41742/301‑1`  
          `tcCTTCATGTTGTCAATACCTGTTTTTCGTGCGACTTATCAGGCTGTCTACTTATCCGGTTGACCCCGGTGTCGATTGGGATGGCTATTCGCCGTGTCCCTCTCGATGGCTGTAAGTATCCTATAGGTTAGACTTTAAGTCAATACTCTTTTTGATCAATTTTAAATTAATCTTTTAAGTCTCTTTAAGTTGTCTCTCCTTATAGTGAGTTGTATTAATAAAGACAATGATTAAAGGTTATCACTTTATGGCTAACAACAAGGCGTCTTTAGGTCTGGTCTTTATGTAG‑TGTCTTTAGGTC                                                                                                                                                                                                                                                                                                                 >  1:345959/3‑301`  
          `gtgtataagagacagATACCTGTTTTTCGTGCGACTTATCAGGCTGTCTACTTATCCGGTTGACCCCGGTGTCGATTGGGATGGCTATTCGCCGTGTCCCTCTCGATGGCTGTAAGTATCCTATAGGTTAGACTTTAAGTCAATACTCTTTTTGATAAATTTTAAATTAATCTTTTAAGTCTCTTTAAGTTGTCTCTCCTTATAGTGAGTTGTATTAATAAAGACAATGATTAAAGGTTATCACTTTATGGCTAACAACAAGGCGTCTTTAGGTCTGGTCTTTATGTAG‑TGTCTTTAGGTC                                                                                                                                                                                                                                                                                                                 <  1:208476/286‑1`  
          `gatgtgtataagagacagCCTGTTTTTCGTGCGACTTATCAGGCTGTCTACTTATCCGGTTGACTCCGGTGTCGATTGGGATGGCTATTCGCCGTGTCCCTCTCGATGGCTGTAAGTATCCTATAGGTTAGACTTTAAGTCAATACTCTTTTTGATAAATTTTAAATTAATCTTTTAAGTCTCTTTAAGTTGTCTCTCCTTATAGGGAGTTGTATTAATAAAGACAATGATTAAAGGTGATCACTTTATGGCTAACAACAAGGCGTCTTTAGGTCTGGTCTTTATGTAG‑TGTCTTTAGGTC                                                                                                                                                                                                                                                                                                                 <  1:29101/283‑1`  
          `TACTTCATGTTGTCAATACCTGTTTTTCGTGCGACTTATCATGCTGTCTACTTATCCGGTTGACCCCGGTGTCGATTGGGATGGCTATTCGCCGTGTCCCTCTCGATGGCTGTAAGTATCCTATAGGTTAGACTTTAAGTCAATACTCTTTTTGATAAATTTTAAATTAATCTTTTAAGACTCTTTAAGTTGTCTCTCCTTATAGTGAGTTGTATTAATAAAGACAATGATTAAAGTTTATCAATTTATGGCTAACAACAAGGCGTCTTTATGTCTGGTCTTTATGTAG‑TGTCTTTAGGTC                                                                                                                                                                                                                                                                                                                 >  1:92405/1‑301`  
          `TACTTCATGTTGTCAATACCTGTTTTTCGTGCGACTTATCAGGCTGTCTACTTATCCGGTTGACCCCGGTGTCGATTGGGATGGCTATTCGCCGTGTCCCTCTCGATGGCTGTAAGTATCCTATAGGTTAGACTTTAAGTCAATACTCTTTTTGATAAATTTTAAATTAATCTTTTAAGTCTCTTTAAGTTGTCTCTCCTTATAGTGAGTTGTATTAATAAAGACAATGATTAAAGGTTATCACTTTATGGCTAACAACAAGGCGTCTTTAGGTCTGGTCTTTATGTAG‑TGTCTTTAGGct                                                                                                                                                                                                                                                                                                                 >  1:191871/1‑299`  
          `TACTTCATGTTGTCAATACCTGTTTTTCGTGCGACTTATCAGGCTGTCTACTTATCCGGTTGACCCCGGTGTCGATTGGGATGGCTATTCGCCGTGTCCCTCTCGATGGCTGTAAGTATCCTATAGGTTAGACTTTAAGTCAATACTCTTTTTGATAAATTTTAAATTAATCTTTTAAGTCTCTTTAAGTTGTCTCTCCTTATAGTGAGTTGTATTAATAAAGACAATGATTAAAGGTTATCACTTTATGGCTAACAACAAGGCGTCTTTAGGTCTGGTCTTTATGTAG‑TGTCTTTAGGTt                                                                                                                                                                                                                                                                                                                 >  1:170017/1‑300`  
          `TACTTCATGTTGTCAATACCTGTTTTTCGTGCGACTTATCAGGCTGTCTACTTATCCGGTTGACCCCGGTGTCGATTGGGATGGCTATTCGCCGTGTCCCTCTCGATGGCTGTAAGTATCCTATAGGTTAGACTTTAAGTCAATACTCTTTTTGATAAATTTTAAATTAATCTTTTAAGTCTCTTTAAGTTGTCTCTCCTTATAGTGAGTTGTATTAATAAAGACAATGATTAAAGGTTATCACTTTATGGCTAACAACAAGGCGTCTTTAGGTCTGGTCTTTATGTAG‑TGTCTTTAGGTC                                                                                                                                                                                                                                                                                                                 <  1:278545/301‑1`  
          `TACTTCATGTTGTCAATACCTGTTTTTCGTGCGACTTATCAGGCTGTCTACTTATCCGGTTGACCCCGGTGTCGATTGGGATGGCTATTCGCCGTGTCCCTCTCGATGGCTGTAAGTATCCTATAGGTTAGACTTTAAGTCAATACTCTTTTTGATAAATTTTAAATTAATCTTTTAAGTCTCTTTAAGTTGTCTCTCCTTATAGTGAGTTGTATTAATAAAGACAATGATTAAAGGTTATCACTTTATGGCTAACAACAAGGCGTCTTTAGGTCTGGTCTTTATGTAG‑TGTCTTTAGGTC                                                                                                                                                                                                                                                                                                                 <  1:167766/301‑1`  
          `TACTTCATGTTGTCAATACCTGTTTTTCGTGCGACTTATCAGGCTGTCTACTTATCCGGTTGACCCCGGTGTCGATTGGGATGGCTATTCGCCGTGTCCCTCTAGCTGGCTGTAAGTCTCCTATAGGTTAGACTTTAAGTCAATACTCTTTTTGATAAATTTTAAATTAATCTTTTAAGTCTCTTTAAGTTGTCTCTCCTTATAGTGAGTTGTATTAATAAAGACAATGATTAACGGTTATCACTTTATGGCTAACAACAAGGCGTCTTTAGGTCTGGTCTTTATGTAG‑TGTCTTTAGGTt                                                                                                                                                                                                                                                                                                                 >  1:211996/1‑300`  
          `TACTTCATGTTGTCAATACCTGTTGTGCGTGCGACTTATCAGGCTGTCTACTTATCCGGTTGACCCCGGTGTCGATTGGGATGGCTATTCGCCGTGTCCCTCTCGATGGCTGTAAGTATCCTATAGGTTAGACTTTAAGTCAATACTCTTTTTGATAAATTTTAAATTAATCTTTTAAGTCTCTTTAAGTTGTCTCTCCTTATAGTGAGTTGTATTAAGAAAGACAATGATTAAAGGTTATCACTTTATGGCTAACAACAAGGCGTCTTTAGGTCTGGTCTTTATGTAG‑TGTCTTTAGGTC                                                                                                                                                                                                                                                                                                                 <  1:46859/301‑1`  
          `TACTTCAGGTTGTTAATACCTGTTTGTCGTGCGACTTATCAGGCTGTCTACTGATCCGGTTGACCCCGGTGTCGATTGGGATGGCTATTCGCAGTGTCCCTCTCGATGGCTGTAAGTATCCTATAGGTTAGACTTTAAGTCAATACTCTTTTTGATAAATTTTAAATTAATCTTGTAAGTCTCTTTAAGTTGTCTCTCCTTATAGTGAGTTGTATTAATAAAGACAATGATTAAAGGTTATCACTTTATGGCTAACAACAAGGCGTCTTTAGGTCTGGTCTTTATGTAG‑TGTCTTTAGGTC                                                                                                                                                                                                                                                                                                                 <  1:57466/301‑1`  
           `ACTTCATGTTGTCAATACCTGTTTTTCGTGCGACTTATCAGGCTGTCTACTTATCCGGTTGACCCCGGTGTCGATTGGGATGGCTATTCGCCGTGTCCCTCTCGATGGCTGTAAGTATCCTATAGGTTAGACTTTAAGTCAATACTCTTTTTGATAAATTTTAAATTAATCTTTTAAGTCTCTTTACGTTGTCTCTCCTTATAGTGCGTTGTATTAATAAAGACAATGATTAAAGGTTATCACTTTATGGCTAACAACAAGGCGTCTTTAGGTCTGGTCTTTATGTAG‑TGTCTTTAGGTCT                                                                                                                                                                                                                                                                                                                >  1:263074/1‑301`  
           `ACTTCATGTTGTCAATACCTGTTTTTCGTGCGACTTATCAGGCTGTCTACTTATCCGGTTGACCCCGGTGTCGATTGGGATGGCTATTCGCCGTGTCCCTCTCGATGGCTGTAAGTATCCTATAGGTTAGACTTTAAGTCAATACTCTTTTTGATAAATTTTAAATTAATCTTTTAAGTCTCTTTAAGTTGTCTCTCCTTATAGTGAGTTGTATTAATAAAGACAATGATTAAAGGTTATCACTTTATGGCTAACAACAAGGCGTCTTTAGGTCTGGTCTTTATGTAT‑TGTCTTTAGTTCT                                                                                                                                                                                                                                                                                                                >  1:205036/1‑301`  
           `ACTTCATGTTGTCAATACCTGTTTTTCGTGCGACTTATCAGGCTGTCTACTTATCCGGTTGACCCCGGTGTCGATTGGGATGGCTATTCGCCGTGTCCCTCTCGATGGCTGTAAGTATCCTATAGGTTAGACTTTAAGTCAATACTCTTTTTGATAAATTTTAAATTAATCTTTTAAGTCTCTTTAAGTTGTCTCTCCTTATAGTGAGTTGTATTAATAAAGACAATGATTAAAGGTTATCACTTTATGGCTAACAACAAGGCGTCTTTAGGTCTGGTCTTTATGTAG‑TGTCTTTAGGTCT                                                                                                                                                                                                                                                                                                                >  1:59705/1‑301`  
            `CTTCATGTTGTCAATACCTGTTTTTCGTGCGACTTATCAGGCTGTCTACTTATCCGGTTGACCCCGGTGTCGATTGGGATGGCTATTCGCCGTGTCCCTCTCGATGGCTGTAAGTATCCTATAGGTTAGACTTTAAGTCAATACTCTTTTTGATAAATTTTAAATTAATCTTTTAAGTCTCTTTAAGTTGTCTCTCCTTATAGTGAGTTGTATTAATAAAGACAATGATTAAAGGTTATCACTTTATGGCTAACAACAAGGCGTCTTTAGGTCTGGTCTTTATGTAG‑TGTCTTTAGctgtc                                                                                                                                                                                                                                                                                                               >  1:155657/1‑296`  
               `tgtataagagacagtTGTTTTTCGTGCGACTTATTAGGCTGTCTACTTATCCGGTTGACCCCGGTGTCGATTGTGATGGCTATTCGCCGTGTCCCTCTCGATGGCTGTAAGTATCCTATAGGTTAGACTTTAAGTCAATACTCTTTTTGATAAATTTTAAATTAATCTTTTAAGTCTCTTTAAGTTGTCTCTCCTTATAGTGAGTTGTATTAATAAAGACAATGATTAAAGGTTATCACTTTATGGCTAACAACAAGGCGTCTTTAGGTCTGGTCTTTATGTAG‑TGTCTTTAGGTCTGGTC                                                                                                                                                                                                                                                                                                            <  1:339956/286‑1`  
               `aagagacagAATACCTGTTTTTCGTGCGACTTATCAGGCTGTCTACTTATCCGGTTGACCCCGGTGTCGATTGGGATGGCTATTCGCCGTGTCCCTCTCGATGGCTGTAAGTATCCTATAGGTTAGACTTTAAGTCAATACTCTTTTTGATAAATTTTAAATTAATCTTTTAAGTCTCTTTAAGTTGTCTCTCCTTATAGTGAGTTGTATTAATAAAGACAATGATTAAAGGTTATCACTTTATGGCTAACAACAAGGCGTCTTTAGGTCTGGTCTTTATGTAG‑TGTCTTTAGGTCTGGTC                                                                                                                                                                                                                                                                                                            <  1:200181/292‑1`  
               `CATGTTGTCAATACCTGTTTTTCGTGCGACTTATCAGGCTGTCTACTTATCCGGTTGACCCCGGTGTCGATTGGGATGGCTATTCGCCGTGTCCCTCTCGATGGCTGTAAGTATCCTATAGGTTAGACTTTAAGTCAATACTCTTTTTGATAAATTTTAAATTAATCTTTTAAGTCTCTTTAAGTTGTCTCTCCTTATAGTGAGTTGTATTAATAAAGACAATGATTAAAGGTTATCACTTTATGGCTAACAACAAGGCGTCTTTAGGTCTGGTCTTTATGTAG‑TGTCTTTAGGTCTGGTC                                                                                                                                                                                                                                                                                                            <  1:332711/301‑1`  
               `CATGTTGTCAATACCTGTTTTTCGTGCGACTTATCAGGCTGTCTACTTATCCGGTTGACCCCGGTGTCGATTGGGATGGCTATTCGCCGTGTCCCTCTCGATGGCTGTAAGTATCCTATAGGTTAGACTTTAAGTCAATACTCTTTTTGATAAATTTTAAATTAATCTTTTAAGTCTCTTTAAGTTGTCTCTCCTTATAGTGAGTTGTATTAATAAAGACAATGATTAAAGGTTATCACTTTATGGCTAACAACAAGGCGTCTTTAGGTCTGGTCTTTATGTAG‑TGTCTTTAGGTCTGGTC                                                                                                                                                                                                                                                                                                            <  1:243346/301‑1`  
                `aagagccagATACCTGTTTTTCGTGCGACTTATCAGGCTGTCTACTTATCCGGTTGACCCCGGTGTCGATTGGGATGGCTATTCGCCGTGTCCCTCTCGATGGCTGTAAGTATCCTATAGGTTAGACTTTAAGTCAATACTCTTTTTGATAAATTTTAAATTAATCTTTTAAGTCTCTTTAAGTTGTCTCTCCTTATAGTGAGTTGTATTAATAAAGACAATGATTAAAGGTTATCACTTTATGGCTAACAACAAGGCGTCTTTAGGTCTGGTCTTTATGTAG‑TGTCTTTAGGTCTGGTCT                                                                                                                                                                                                                                                                                                           <  1:60638/292‑1`  
                `ATGTTGTCAATACCTGTTTTTCGTGCGACTTATCAGGCTGTCTACTTATCCGGTTGACCCCGGTGTCGATTGGGATGGCTATTCGCCAAGTCCCTCTCGATGGCTGTAAGTATCCTATAGGTTAGACTTTAAGTCAATACTCATTTTGATAAATTTTAAATTAATCTTTTAAGTCTCTTTAAGTTGTCTCTCCTTATAGTGAGTTGTATTAATAAAGACAATGATTAAAGGTTATCACTTTATGGCTAACAACAAGGCGTCTTTAGGTCTGGTCTTTATGTAG‑TGTCTTTAGGTCTGGTCT                                                                                                                                                                                                                                                                                                           >  1:144334/1‑301`  
                `ATGTTGTCAATACCTGTTTTTCGTGCGACCTATCAGGCTGTCTACTTATCCGGTTGACCCCGGTGTCGATTGGGATGGCTATTCGCCGTGTCCCTCTCGATGGCTGTAAGTATCCTATAGGTTAGACTTTAAGTCAATACTCTTTTTGATAAATTTTAAATTAATCTTTTAAGTCTCTTTAAGTTGTCTCTCCTTCTAGTGAGTTGTATTAATAAAGACAATGATTAAAGGTTATCACTTTATGGCTACCAACAAGGCGTCTTTAGGTCTGGTCTTTATGTCG‑TGTCTTTAGGTCTGTTCT                                                                                                                                                                                                                                                                                                           >  1:256364/1‑301`  
                     `GTCAATACCTGTTTTTCGTGCGACTTATCAGGCTGTCTACTTATCCGGTTGACCCCAGTGTCGATTGGTATGGCTATTCGCCGTGTCCCTCTCGATGGCTGTAAGTATCCTATAGGTTAGACTTTAAGTCAATACTCCTTTTGATAAATTTTAAATTAATCTTTTAAGTCTCTTTAAGTTGTCTCTCCTTATAGTGAGTTGTATTAATAAAGACAATGATTAAAGGTTATCACTTTATGGCTAACAACAAGGCGTCTTTAGGTCTGGTCTTTATGTAG‑TGTCTTTAGGTCTGGTCTTTtgg                                                                                                                                                                                                                                                                                                      >  1:284438/1‑298`  
                       `ccctccctttttttctttctccttctcctGCTTTCTCCTTCTCCGGTTGCCCCCGGTGTCGATTGGGATGGCTATTCGCCGTGTCCCTCTCGATGGCTGTAAGTATCCTATAGGTTAGACTTTAAGTCAATACTCTTTTTGATAAATTTTAAATTAATCTTTTAAGTCTCTTTAAGTTGTCTCTCCTTATAGTGAGTTGTATTAATAAAGACAATGATTAAAGGTTATCACTTTATGGCTAACAACAAGGCGTCTTTAGGTCTGGTCTTTATGTAG‑TGTCTTTAGGTCTGGGCTTTAGGTc                                                                                                                                                                                                                                                                                                    >  1:249333/30‑300`  
                                                        `TCTAATTATCCGGTGGACCCCGGTGTCGATTGGGATGGCTATTCGCCGTGTCCCTCTCGATGGCTGTAAGTATCCTATAGGTTAGACTTTAAGTCAATACTCTTTTTGATAAATTTTAAATTAATCTTTTAAGTCTCTTTAAGTTGTCTCTCCTTATAGTGAGTTGTATTAATAAAGACAATGATTAAAGGTTATCACTTTATGGCTAACAACAAGGCGTCTTTAGGTCTGGTCTTTATGTAG‑TGTCTTTAGGTCTGGTCTTTATGTTTAAACTTTAAGATAGGCGTTGACTTGATGGGTC                                                                                                                                                                                                                                                                   <  1:344643/301‑1`  
                                                                                                                                                                                                    `AAGTTGTCTCTCCTTATAGTGAGTTGTATTAATAAAGACAATGATTAAAGGTTATCACTTTATGGCTAACAACAAGGCGTCTTTAGGTCTGGTCTTTATGTAG‑TGTCTTTAGGTCTGGTCTTTATGTTTAAACTTTAAGATAGGCGTTGACTTGATGGGTCTTTAGGTGTAGGCTTTAGGTGTTGGCTTTAGGATGGACGTTAGGAGGTGACTTTAGGAGGATACTTTAGGAGACTGTAACAGATAGGGACACAGAGAGACACTCAAGGTAACACCCAAAGACAAACCCAAGGGTCATCCC                                                                                                                       <  1:81724/301‑1`  
                                                                                                                                                                                                                                                                               `GGCGTCTTTAGGTCTGGTCTTTATGTAG‑TGTCTTTAGGTCTGGTCTTTATGTTTAAACTTTAAGATAGGCGTTGACTTGATGGGTCTTTAGGTGTAGGCTTTAGTTGTTGGCTTTAGGATGGACGTTAGGAGGTGACTTTAGGAGGATACTTTAGGAGACTGTAACAGATAGGGACACAGAGAGACACTCAAGGTAACACCCAAAGACAAACCCAAGGGTCATCCCCAACCCTTAGGGAACCCTCAAGGTCAACCGAAGGTTGACTTTAGGTGATTGGCTGGGCTTTAGGTACCCCCTATG                                            <  1:21970/301‑1`  
                                                                                                                                                                                                                                                                                        `ccggagatgtgtataagagacagCTTTAGGTCTGGTCTTTATGTTTAAACTTTAAGATAGGCGTTGACTTGATGGGTCTTTAGGTGTAGGCTTTAGGTGTTGGCTTTAGGATGGACGTTAGGAGGTGACTTTAGGAGGATACTTTAGGAGACTGTAACAGATAGGGACACAGAGAGACACTCAAGGTAACACCCAAAGACAAACCCAAGGGTCATCCCCAACCCTTAGGGAACCCTCAAGGTCAACCGAAGGTTGACTTTAGGTGATTGGCTGGGCTTTAGGTACCCCCTATGGGGGAACT                                    <  1:142125/278‑1`  
                                                                                                                                                                                                                                                                                             `taagcgacatgttctTGTCTTTAGGTCTGGTCTTTATGTTTAAACTTTAAGATAGTCGTTTACTTGATGTGTCTTTAAGTGTAGGCTTTATGTGTTGGCTTTAGGATGGACGTTAGGAGGTGACTTTAGGAGGATACTTTAGGAGACTGTAACAGATGGGGACACAGAGAGACACTCAAGGTAACACCCAAAGACAAACCCAAGGGTCATCCCCAACCCTTAGGGAACCCTCAAGGTCGACCGAAGGTTGACTTTAGGTGATTGGCTGTGCTTTAGGTACCCCCTATGGGGGAACTTTAGG                               <  1:158442/286‑1`  
                                                                                                                                                                                                                                                                                              `atgtgtataagagacagCTTTAGGTCTGGTCTTTATGTTTAAACTTTAAGATAGGCGTTGACTTGATGGGTCTTTAGGTGTAGGCTTTAGGTGTTGGCTTTAGGATGGACGTTAGGAGGTGACTTTAGGAGGATACTTTAGGAGACTGTAACAGATAGGGACACAGAGAGACACTCAAGGTAACACCCAAAGACAAACCCAAGGGTCATCCCCAACCCTTA‑GGAACCCTCAAGGTCAACCGAAGGTTGACTTTAGGTGATTGGCTGGGCTTTAGGTACCCCCTATGGGGGAACTTTAGGTC                             <  1:314551/284‑1`  
                                                                                                                                                                                                                                                                                               `tgtgtataagagacagccTTAGGTCTGGTCTTTAAGTTTAAACTTTAAGATAGGCGTTGACTTGATGGGTCTTTAGGTGTAGGCTTTAGGTGTTGGCTTTAGGATGGACGTTAGGAGGTGACTTTAGGAGGATACTTTAGGAGACTGTAACAGATAGGGAAACAGAGAGACACTCAAGGTAACACCCAAAGACAAACCCAAGGGTCATCCCCAACCCTTAGGGAACCCTCAAGGTCAACCGAAGGTTGACTTTAGGTGATTGGCTGGGCTTTAGGTACCCCCTATGGGGGAACTTTAGGTC                             <  1:315040/283‑1`  
                                                                                                                                                                                                                                                                                               `tgtgtataagagacagCTTTAGGTCTGGGCTTTATGTTTAAGCTTTAAGATAGGCGTTGACTTGATGGGTCTTTAGGTGTAGGCTTTAGGTGTTGGCTTTAGGATGGACGTTAGGAGGTGACTTTAGGAGGATACTTTAGGAGACTGTAACAGATAGGGACACAGAGAGACACTCAAGGTAACACCCAAAGACAAACCCAAGGGTCATCCCCAACCCTTAGGGAACCCTCAAGGTCAACCGAAGGTTGACTTTAGGTGATTGGCTGGGCTTTAGGTACCCCCTATGGGGGAACTTTAGGTC                             <  1:232108/285‑1`  
                                                                                                                                                                                                                                                                                                    `ttatggctgtTCTTTAGGTCTGGTTTTTATGTTTAGACTTTAAGATAGGCGTTGACTTGATGGGTCTTTAGGTGTTGGCTTTAGGTGTTGGCTTTAGGCTGGACGGTAGGAGGTGACTTTAGGAGGATACTTTAGGAGACTTTAACAGATAGGGACACAGAGAGACACTCAAGGTAACACCCAAAGACAAACCCAAGGGTCATCCCCAACCCTTAGGGAACCCTCAAGGTCAACCGAAGGTTGACTTTAGGTGATTGGCTGGGCTTTAGGTACCCCCTATGGGGGAACTTTAGGTCCGTAC                        <  1:288828/291‑1`  
                                                                                                                                                                                                                                                                                                    `ttaggtttgtgtTTTAGTTCTGGTTGTTATTTTTAAACTTTAAGATAGGCGTTGACTTGATGGGTCTTTAGGTGTAGGCTTTAGGTGTTGGCTTTAGGATGGACGTTAGGAGGTGACTTTAGGAGGATACTTTAGGAGACTGTAACAGATAGGGACACAGAGAGACACTCAAGTTAACACCCAAAGACAAACCCAAGGGTCATCCCCAACCCTTAGGGAACCCTCAAGGTCAACCGAAGGTTGACTTTAGGTGATTGGCTGGGCGTTAGGTACCCCCTATGGGGGAACTTTAGGTCCGTAC                        <  1:63643/289‑1`  
                                                                                                                                                                                                                                                                                                    `gggagatttgtataagagacaGGTCTTTATGTTTAAACTTTAAGATAGGCGTTGACTTGATGGGTCTTTAGGTGTAGGCTTTAGGTGTTGGCTTTAGGATGGACGTTAGGAGGTGACTTTAGGAAGATACTTTAGGAGACTGTAACAGATAGGGACACAGAGAGACACTCAAGGTAACACCCAAAGACAAACCCAAGGGTCATCCCCAACCCTTAGGGAACCCTCAAGGTCAACCGAAGGTTGACTTTAGGTGATTGGCTGGGCTTTAGGTACCCCCTATGGGGGAACTTTAGGTCCGTAC                        <  1:215221/280‑1`  
                                                                                                                                                                                                                                                                                                    `ggagatgtgtataagagacagGGTCTTTATGTTTAAACTTTAAGATAGTCGTTGACTTGATGGGTCTTTAGGTGTAGGCTTTAGGTGTTGGCTTTAGGATGGACGTTAGGAGGTGACTTTAGGAGGATACTTTAGGAGACTGTAACAGATAGGGACACAGAGAGACACTCAAGGTAACACCCAAAGACAAACCCAAGGGTCATCCCCAACCCTTAGGGAACCCTCAAGGTCAACCGAAGGTTGACTTTAGGTGATTGGCTGGGCTTTAGGTACCCCCTATGGGGGAACTTTAGGTCCGTAC                        <  1:267634/280‑1`  
                                                                                                                                                                                                                                                                                                    `cggagatgagtataagagacaGGTCTTTATGTTTAAACTTTAAGATAGGCGTTGACTTGATGGGGCTTTAGGTGTAGGCTTTAGGGGTTGGCTTTAGGATTGACGGTAGGAGGTGACTTTAGGAGGATACTTTAGGAGACTGGAACAGATAGGGACACAGAGAGACACTCAAGGTAACACCCAAAGACAAACCCAAGGGTCATCCCCAACCCTTAGGGAACCCTCAAGGTCAACCGAAGGTTGACTTTAGGTGATTGGCTGGGCTTTAGGTACCCCCTATGGGGGAACTTTAGGTCCGTAC                        <  1:106134/280‑1`  
                                                                                                                                                                                                                                                                                                      `tgtataagagaccgGGTCTGGTCTTTATGTTTAAACTTTAAGATAGGCGTTGACTTGATGGGTCTTTAGGTGTAGGCTTTAGGTGTTGGCTTTAGGATGGACGTTAGGAGGTGACTTTAGGAGGATACTTTAGGAGACTGTAACAGATAGGGACACAGAGAGACACTCAAGGTAACACCCAAAGACAAACCCAAGGGTCATCCCCAACCCTTAGGGAACCCTCAAGGTCAACCGAAGGTTGACTTTAGGTGATTGGCTGGGCTTTAGGTACCCCCTATGGGAGAACTTTAGGTCCGTACAC                      <  1:56340/287‑1`  
                                                                                                                                                                                                                                                                                                      `tgtataagagacagGGTCTGGTCTTTATGTTTAAACTTTAAGATAGGCGTTGACTTGATGGGTCTTTAGGTGTAGGCTTTAGGTGTTGGCTTTAGGATGGACGTTAGGAGGTGACTTTAGGAGGATACTTTAGGAGACTGTAACAGATAGGGACACAGAGAGACACTCAAGGTAACACCCAAAGACAAACCCAAGGGTCATCCCCAACCCTTAGGGAACCCTCAAGGTCAACCGAAGGTTGACTTTAGGTGATTGGCTGGGCTTTAGGTACCCCCTATGGGGGAACTTTAGGTCCGTACAC                      <  1:105101/287‑1`  
                                                                                                                                                                                                                                                                                                      `tgtataagagacagGGTCTGGTCTTTATGTTTAAACTTTAAGATAGGCGTTGACTTGATGGGTCTTTAGGTGTAGGCTTTAGGTGTTGGCTTTAGGATGGACGTTAGGAGGTGACTTTAGGAGGATACTTTAGGAGACTGTAACAGATAGGGACACAGAGAGACACTCAAGGTAACACCCAAAGACAAACCCAAGGGTCATCCCCAACCCTTAGGGAACCCTCAAGGTCAACCGAAGGTTGACTTTAGGTGATTGGCTGGGCTTTAGGTACCCCCTATGGGGGAACTTTAGGTCCGTACAC                      <  1:304941/287‑1`  
                                                                                                                                                                                                                                                                                                      `gagatgtgtataagagaaaGGTCTTTATGTTTAAACTTTAAGATAGGCGTTGACTTGATGGGTCTTTAGGTGTAGGCTTTAGGTGTTGGCTTTAGGATGGACGTTAGGAGGTGACTTTAGGAGGATACTTTAGGAGACTGTAACAGATAGGGACACAGAGAGACACTCAAGGTAACACCCAAAGACAAACCCAAGGGTCATACCCAACCCTTAGGGAACCCTCAAGGTCAACCGAAGGTTGACTTTAGGTGATTGGCTGGGCTTTAGGTACCCCCTATGGGGGAACTTTAGGTCCGTACAC                      <  1:67282/282‑1`  
                                                                                                                                                                                                                                                                                                      `atgtctTGTCTTTAGGTCTGGTCTTTATGTTTAAACTGTAAGATAGTCGTTGACTTGATGGGTCTTTAGGTGTAGGCTTTAGGTGTTGGCTTTAGGATGGACGTTAGGAGGTGACTTTAGGAGGATACTTTAGGAGACTGTAACAGATAGGGACACAGAGAGACACTCAAGGTAACACCCAAAGACAAACCCAAGGGTCATCCCCAACCCTTAGGGAACCCTCAAGGTCAACCGAAGGTTGACTTTAGGTGATTGGCTGGGCTTTAGGTACCCCCTATGGGGGAACTTTAGGTCCGTACAC                      <  1:19805/295‑1`  
                                                                                                                                                                                                                                                                                                      `aggtttTGTCTTTAGGTCTGGTCTTTATGTTTAAACTTTAAGATAGGCGTTGACTTGATGGGTCTTTAGGTGTAGGCTTTGGGTGTTGGCTTTAGGATGGACGTTAGGAGGTGACTTTAGGAGGATACTTTAGGAGACTGTAACAGATAGGGACACAGAGAGACACTCAAGGTAACACCCAAAGACAAACCCAAGGGTCATCCCCAACCCTTAGGGAACCCTCAAGGTCAACCGAAGGTTGACTTTAGGTGATTGGCTGGGCTTTAGGTACCCCCTATGGGGGAACTTTAGGTCCGTACAC                      <  1:347098/295‑1`  
                                                                                                                                                                                                                                                                                                      `agatgtgtataagagacagGGTCTTTATGTTTAAACTTTAAGATAGGCGTTGACTTGATGGGTCTTTAGGTGTAGGCTTTAGGTGTTGGCTTTAGGATGGACGTTAGGAGGTGACTTTAGGAGGATACTTTAGGAGACTGTAACAGATAGGGACACAGAGAGACACTCAAGGTAACACCCAAAGACAAACCCAAGGGTCATCCCCAACCCTTAGGGAACCCTCAAGGTCAACCGAAGGTTGACTTTAGGTGATTGGCTGGGCTTTAGGTACCCCCTATGGGGGAACTTTAGGTCCGTACAC                      <  1:103695/282‑1`  
                                                                                                                                                                                                                                                                                                      `aagcgacagCTTTAGGTCTGGTCTTTATGTTTAAACTTTAAGATAGGCGTTGACTTAATGGGTCTTTAGGTGCAGGCTTTAGGTGTTGGCTTTAGGATGGACGTTAGGAGGTGACTTTAGGAGGATACTTTATGAGACTGTAACAGATAGGGACACAGAGAGACACTCAAGGTAACACCCAAAGACAAACCCAAGGGTCATCCCCAACCCTTAGGGAACCCTCAAGGTCAACCGAAGGTTGACTTTAGGTGATTGGCTGGGCTTTAGGTACCCCCTATGGGGGAACTTTAGGTCCGTACAC                      <  1:90093/292‑1`  
                                                                                                                                                                                                                                                                                                      `aagagacagCTTTAGGTCTGGTCTTTATGTTTAAACTTTAAGATAGGCGTTGACTTGATGGGTCTTTAGGTGTAGGCTTTAGGTGTTGGCTTTAGGATGGACGTTAGGAGGTGACTTTAGGAGGATACTTTAGGAGACTGTAACAGGTAGGGACACAGAGAGACACTCAAGGTAACACCCAAAGACAAACCCAAGGGTCATCCCCAACCCTTAGGGAACCCTCAAGGTCAACCGAAGGTTGACTTTAGGTGATTGGCTGGGCTTTAGGTACCCCCTATGGGGGAACTTTAGGTCCGTACAC                      <  1:24083/292‑1`  
                                                                                                                                                                                                                                                                                                      `aagagacagCTTTAGGTCTGGTCTTTATGTTTAAACTTTAAGATAGGCGTTGACTTGATGGGTCTTTAGGTGTAGGCTTTAGGTGTTGGCTTTAGGATGGACGTTAGGAGGTGACTTTAGGAGGATACTTTAGGAGACTGTAACAGATAGGGACACAGAGAGACACTCAAGGTAACACCCAAAGACAAACCCAAGGGTCATCCCCAACCCTTAGGGAACCCTCAAGGTCAACCGAAGGTTGACTTTAGGTGATTGGCTGGGCTTTAGGTACCCCCTATGGGGGAACTTTAGGTCCGTACAC                      <  1:245232/292‑1`  
                                                                                                                                                                                                                                                                                                      `aagagacagCTTTAGGTCTGGTCTTTATGTTTAAACTTTAAGATAGGCGTTGACTTGATGGGTCTTTAGGTGTAGGCTTTAGGTGTTGGCTTTAGGATGGACGTTAGGAGGTGACTTTAGGAGGATACTTTAGGAGACTGTAACAGATAGGGACACAGAGAGACACTCAAGGTAACACCCAAAGACAAACCCAAGGGTCATCCCCAACCCTTAGGGAACCCTCAAGGTCAACCGAAGGTTGACTTTAGGTGATTGGCTGGGCTTTAGGTACCCCCTATGGGGGAACTTTAGGTCCGTACAC                      <  1:77976/292‑1`  
                                                                                                                                                                                                                                                                                                      `aagagacagCTTTAGGTCTGGTCTTTATGTTTAAACTTTAAGATAGGCGTTGACTTGATGGGTCTTTAGGTGTAGGCTTTAGGTGTTGGCTTTAGGATGGACGTTAGGAGGTGACTTTAGGAGGATACTTTAGGAGACTGTAACAGATAGGGACACAGAGAGACACTCAAGGTAACACCCAAAGACAAACCCAAGGGTCATCCCCAACCCTTAGGGAACCCTCAAGGTCAACCGAAGGTTGACTTTAGGTGATTGGCTGGGCTTTAGGTACCCCCTATGGGGGAACTTTAGGTCCGTACAC                      <  1:317587/292‑1`  
                                                                                                                                                                                                                                                                                                      `aagagacagCTTTAGGTCTGGTCTTTATGTTTAAACTTTAAGATAGGCGTTGACTTGATGGGTCTTTAGGTGTAGGCTTTAGGTGTTGGCTTTAGGATGGACGTTAGGAGGTGACTTTAGGAGGATACTTTAGGAGACTGTAACAGATAGGGACACAGAGAGACACTCAAGGTAACACCCAAAGACAAACCCAAGGGTCATCCCCAACCCTTAGGGAACCCTCAAGGTCAACCGAAGGTTGACTTTAGGTGATTGGCTGGGCTTTAGGTACCCCCTATGGGGGAACTTTAGGTCCGTACAC                      <  1:225453/292‑1`  
                                                                                                                                                                                                                                                                                                      `aagagacagCTTTAGGTCTGGTCTTTATGTTTAAACTTTAAGATAGGCGTTGACTTGATGGGTCTTTAGGTGTAGGCTTTAGGTGTTGGCTTTAGGATGGACGTTAGGAGGTGACTTTAGGAGGATACTTTAGGAGACTGTAACAGATAGGGACACAGAGAGACACTCAAGGTAACACCCAAAGACAAACCCAAGGGTCATCCCCAACCCTTAGGGAACCCTCAAGGTCAACCGAAGGTTGACTTTAGGTGATTGGCTGGGCTTTAGGTACCCCCTATGGGGGAACTTTAGGTCCGTACAC                      <  1:294354/292‑1`  
                                                                                                                                                                                                                                                                                                      `aagagacagCTTTAGGTCTGGTCTTTATGTTTAAACTTTAAGATAGGCGTTGACTTGATGGGACTTTAGGTGTAGGCTTTAGGTGTTAGCTTTAGGATGGACGTTAGGAGGTGACGTTAGGAGGATACTTTAGGAGACTGTAACAGATAGGGACACAGAGAGACACTCAAGGTAACACCCAAAGACAAACCCAAGGGTCATCCCCAACCCTTAGGGAACCCTCAAGGTCAACCGAAGGTTGACTTTAGGTGATTGGCTGGGCTTTAGGTACCCCCTATGGGGGAACTTTAGGTCCGTACAC                      <  1:184903/292‑1`  
                                                                                                                                                                                                                                                                                                             `GTCTTTAGGTCTGGTCTTTATGTTTAAACTTTAAGATAGGCGTTGACTTGATGGGTCTTTAGGTGTAGGCTTTAGGTGTTGGCTTTAGGATGGACGTTAGGAGGTGACTTTAGGAGGATACTTTAGGAGACTGTCACAGCTAGGGACACAGAGAGACACTCAAGGTAACACCCAAAGACAAACCCAAGGGTCATCCCCAACCCTTATGGAACCCTCAAGGTCAACCGAAGGTTGACTTTAGGTGATTGGCTGGGCTTTAGGTACCCCCTATGGTGGAACTTTAGGTCCGTACACctgtctc               >  1:222184/1‑294`  
                                                                                                                                                                                                                                                                                                             `GTCTTTAGGTCTGGTCTTTATGTTTAAACTTTAAGATAGGCGTTGACTTGATGGGTCTTTAGGTGTAGGCTTTAGGTGTTGGCTTTAGGATGGACGTTAGGAGGTGACTTTAGGAGGATACTTTAGGAGACTGTAACAGATCGGGACACAGAGAGACACTCAAGGTAACACCCAAAGACAAACCCAAGGGTCATCCCCAACCCTTAGGGAACCCTCAAGGTCAACCGAAGGTTGACTTTAGGTGATTGGCTGGGCTTTAGGTACCCCCTATGGGGGAACTTTAGGTCCGTACctgtctctt               >  1:265393/1‑292`  
                                                                                                                                                                                                                                                                                                             `GTCTTTAGGTCTGGTCTTTATGTTTAAACTTTAAGATAGGCGTTGACTTGATGGGTCTTTAGGTGTAGGCTTTAGGTGTTGGCTTTAGGATGGACGTTAGGAGGTGACTTTAGGAGGATACTTTAGGAGACTGTAACAGATAGGGACACAGAGAGACACTCAAGGTAACACCCAAAGACAAACCCAAGGGTCATCCCCAACCCTTAGGGAACCCTCACGGTCAACCGAAGGTTGACTTTAGGGGATTGGCTGGGCTTTAGGTACCCCCTATGGGGGAACTTTAGGTCCGTACACctgtatt               >  1:73743/1‑294`  
                                                                                                                                                                                                                                                                                                             `GTCTTTAGGTCTGGTCTTTATGTTTAAACTTTAAGATAGGCGTTGACTTGATGGGTCTTTAGGTGTAGGCTTTAGGTGTTGGCTTTAGGATGGACGTTAGGAGGTGACTTTAGGAGGATACTTTAGGAGACTGTAACAGATAGGGACACAGAGAGACACTCAAGGTAACACCCAAAGACAAACCCAAGGGTCATCCCCAACCCTTAGGGAACCCTCAAGGTCAACCGAAGGTTGACTTTAGGTGATTGGCTGTGCTTTAGGTACCCCCTATGGGGGAACTTTAGGTCCGTACctgtctctt               >  1:261559/1‑292`  
                                                                                                                                                                                                                                                                                                             `GTCTTTAGGTCTGGTCTTTATGTTTAAACTTTAAGATAGGCGTTGACTTGATGGGTCTTTAGGTGTAGGCTTTAGGTGTTGGCTTTAGGATGGACGTTAGGAGGTGACTTTAGGAGGATACTTTAGGAGACTGTAACAGATAGGGACACAGAGAGACACTCAAGGTAACACCCAAAGACAAACACAAGGGTCATCCCCAACCCTTAGGGAACCCTCAAGGTCAACCGAAGGTTGACTTTAGGTGATTGGATGGGCTTTAGGTACCCCCTATGGGGGAACTTTAGGTCCGTACACctgtctc               >  1:117960/1‑294`  
                                                                                                                                                                                                                                                                                                               `CTTTATGTCTGGTCTTTATGTTTAAACTTTAAGATAGGCGTTGACTTGATGGGTCTTTAGGTGTAGGCTTTAGGTGTTGGCTTTAGGATGGACGTTAGGAGGTGACTTTAGGAGGATACTTTAGGAGACTGTAACAGATAGGGACACAGAGAGACACTCAAGGTAACACCCAAAGACAAACCCAAGGGTCATCCCCAACCCTTAGGGAACCCTCAAGGTCAACCGAAGGTTGACTTTAGGTGATTGGCTGGGCTTTAGGTACCCCCTATGGGGGAACTTTAGGTCCGTACACTGTGAGAta             >  1:14752/1‑299`  
                                                                                                                                                                                                                                                                                                               `CTTTAGGTCTGGTCTTTATGTTTAAACTTTAAGATAGTCGTTGACTTGATGGGTCTTTAGGTGTAGGCTTTAGGTGTTGGCTTTAGGATGGAAGTTAGGAGGTGACTTTAGGCGGATACTTTCGGAGACTGTAACAGATAGGGACACAGAGAGACACACAAGGTACCACCCAAAGACCAACCCAAGGGTCATCCCCACCCCTTAGGGAACCCTCAAGGTCAACCGAAGGTTGACTTTAGGTGATTGGCTGGGCTTTAGGTACCCCCTATGGGGGCACTTTAGGTCCGTACACctgtctctt             >  1:151479/1‑292`  
                                                                                                                                                                                                                                                                                                               `CTTTAGGTCTGGTCTTTATGTTTAAACTTTAAGATAGGCGTTGACTTGATGGGTCTTTAGGTGTAGGCTTTAGGTGTTGGCTTTAGGATGTACGTTAGGAGGTGACTTTAGGAGGATACTTTAGGAGACTGTAACAGATAGGGACACAGAGAGACACTCAAGGTAACACCCAAAGACAAACCCAAGGGTCATCCCCAACCCTTAGGGAACCCTCAAGGTCAACCGAAGGTTGACTTTAGGTGAATGGCTGGGCTTTAGGTACCCCCTATGGGGGAACTTTAGGTCCGTACACTGTctgtct             >  1:301586/1‑295`  
                                                                                                                                                                                                                                                                                                               `CTTTAGGTCTGGTCTTTATGTTTAAACTTTAAGATAGGCGTTGACTTGATGGGTCTTTAGGTGTAGGCTTTAGGTGTTGGCTTTAGGATGGACGTTAGGAGGTGACTTTAGGAGGATACTTTAGGAGACTGTAACAGATAGGGACACAGAGAGACACTCAAGGTAACACCCAAAGACAAACCCAAGGGTCATCCCCAACTCTTAGGGAACCCTCAAGGTCAACCGAAGGCTGGCTTTAGGTGATTGGCTGGGCCTGAGGTAACCCATATGGCGGAACTTTAGGTCCGTTCACggatctaac             >  1:72711/1‑292`  
                                                                                                                                                                                                                                                                                                               `CTTTAGGTCTGGTCTTTATGTTTAAACTTTAAGATAGGCGTTGACTTGATGGGTCTTTAGGTGTAGGCTTTAGGTGTTGGCTTTAGGATGGACGTTAGGAGGTGACTTTAGGAGGATACTTTAGGAGACTGTAACAGATAGGGACACAGAGAGACACTCAAGGTAACACCCAAAGACAAACCCAAGGGTCATCCCCAACCCTTAGGGAACCCTCAAGGTCAACCGAAGGTTGACTTTAGGTTATTGGCTGGGCTTTAGGTACCCCCTATGGGGGAACTTTAGGTCCGTACctgtctcttat             >  1:346120/1‑290`  
                                                                                                                                                                                                                                                                                                               `CTTTAGGTCTGGTCTTTATGTTTAAACTTTAAGATAGGCGTTGACTTGATGGGTCTTTAGGTGTAGGCTTTAGGTGTTGGCTTTAGGATGGACGTTAGGAGGTGACTTTAGGAGGATACTTTAGGAGACTGTAACAGATAGGGACACAGAGAGACACTCAAGGTAACACCCAAAGACAAACCCAAGGGTCATCCCCAACCCTTAGGGAACCCTCAAGGTCAACCGAAGGTTGACTTTAGGTGATTGGCTGGGCTTTAGGTACCCCCTATGGGGGAACTTTAGGTactgtctcttatacaca             >  1:190838/1‑284`  
                                                                                                                                                                                                                                                                                                               `CTTTAGGTCTGGTCTTTATGTTTAAACTTTAAGATAGGCGTTGACTTGATGGGTCTTTAGGTGTAGGCTTTAGGTGTTGGCTTTAGGATGGACGTTAGGAGGTGACTTTAGGAGGATACTTTAGGAGACTGTAACAGATAGGGACACAGAGAGACACTCAAGGTAACACCCAAAGACAAACCCAAGGGTCATCCCCAACCCTTAGGGAACCCTCAAGGTCAACCGAAGGTTGACTTTAGGTGATTGGCTGGGCTTTAGGTACCCCCTATGGGGGAACTTTAGGTCCtgtctcttatacaca             >  1:130809/1‑286`  
                                                                                                                                                                                                                                                                                                               `CTTTAGGTCTGGTCTTTATGTTTAAACTTTAAGATAGGCGTTGACTTGATGGGTCTTTAGGTGTAGGCTTTAGGTGTTGGCTTTAGGATGGACGTTAGGAGGTGACTTTAGAAGGATACTTTAGGAGACTGTAACAGATAGGGACACAGAGAGACACTCAAGGTAACAACCAAAGACAAACCCAAGGGTCATCCCCAACCCTTAGGGAACCCTCAAGGTCAACCGAAGGTTGACTTTAGGTGATTGGCTGGGCTTTAGGTACCCCCTATGGGGGAACTTTAGGTCCGTACccctgtctctt             >  1:175768/1‑290`  
                                                                                                                                                                                                                                                                                                                   `AGGTCTGGTCTTTATGTTTAAACTTTAAGATAGGCGTTGACTTGATGGGTCTTTAGGTGTAGGCTTTAGGTGTTGGCTTTAGGATGGACGTTAGGAGGTGACTTTAGGAGGATACTTTAGGAGACTGTAACAGATAGGGACACAGAGAGACACTCAAGGTAACACCCAAAGACAAACCCAAGGGTCATCCCCAACCCTTAGGGAACCCTCAAGGTCAACCGAAGGTTGACTTTAGGTGATTGGCTGGGCTTTAGGTACCCCCTATGGGGGAACTTctgtctcttatacacatctccgagcc         >  1:298109/1‑275`  
                                                                                                                                                                                                                                                                                                                    `GGTCTGGTCTTTATGTTTAAACTTTAAGATAGGCGTTGACTTGATGGGTCTTTAGGTGTAGGCTTTAGGTGTTGGCTTTAGGATGGACGTTAGGAGGTGACTTTAGGAGGATACTTTAGGAGACTGTAACAGATATGGACACAGAGAGACACTCAAGGTAACACCCAAAGACAAACCCAAGGGTCATCCCCAACCCTTAGGGAACCCTCAAGGTCAACCGAAGGTTGACTTTAGGTGATTGGCTGGGCTTTAGGTACCCCCTATGGGGGAACTTTAGGTCCtgtctcttatacacatctcc        >  1:286934/1‑281`  
                                                                                                                                                                                                                                                                                                                    `GGTCTGGTCTTTATGTTTAAACTTTAAGATAGGCGTTGACTTGATGGGTCTTTAGGTGTAGGCTTTAGGTGTTGGCTTTAGGATGGACGTTAGGAGGTGACTTTAGGAGGATACTTTAGGAGACTGTAACAGATAGGGACACAGAGAGACACTCAAGGTAACACCCAAAGACAAACCCAAGGGTCATCCCCAACCCTTAGGGAACTCTCAAGGTCAACCGAAGGTTGACTTTAGGTGATTGGCTGGGCTTTAGGTACCCCCTCTGGGGGAACTTTAGGTactgtctcttatacccctctcc        >  1:181798/1‑279`  
                                                                                                                                                                                                                                                                                                                     `GTCTGGTCTTTATGTTTAAACTTTAAGATAGGCGTTGATTTGATGGGTCTTTAGGTGTAGGCTTTAGGTGTTGGCTTTAGGATGGACGTTAGGAGGTGACTTTAGGAGGATACTTTAGGAGACTGTAACAGATAGGGACCCAGAGAGACACTCAAGGTAACACCCAAAGACAAACCCAAGGGTCATCCCCAACCCTTAGGGAACCCTCAAGGTCAACCGAAGGTTGACTTTAGGTGATTGGCTGGGCTTTAGGTACCCCCTATGGGGGAACTTTAGGTCCGTACACctgtctcttatacac       >  1:150183/1‑286`  
                                                                                                                                                                                                                                                                                                                         `GGTCTTTATGTTTAAACTTTAAGATAGGCGTTGACTTGATGGGTCTTTAGGTGTAGGCTTTAGGTGTTGGCTTTAGGATGGACGTTAGGAGGTGACTTTAGGAGGATACTTTAGGAGACTGTAACAGATAGGGACACAGAGAGACACTCAAGGTAACACCCAAAGACACACCCAAGGGTCATCCCCAACCCTTAGGGAACCCTCAAGGTCAACCGAAGGTTGACTTTAGGTGATTGGCTGGGCTTTATGTACCCCCTATGGGGGAACTTTAGGTCCGTACACctgtctcttatacaaatct   >  1:172433/1‑282`  
                                                                                                                                                                                                                                                                                                                         `GGTCTTTATGTTTAAACTTTAAGATAGGCGTTGACTTGATGGGTCTTTAGGTGTAGGCTTTAGGTGTTGGCTTTAGGATGGACGTTAGGAGGTGACTTTAGGAGGATACTTTAGGAGACTGTAACAGATAGGGACACAGAGAGACACTCAAGGTAACACCCAAAGACAAACCCAAGGGTGATCCCCAACCCTTAGGGAACCCTCAAGGTCAACCGAAGGTTGACTTTAGGTGATTGGCTGGGCTTTAGGTACCCCCTATGGTTGAACTTTAGGTCCGTACACctgtctcttatacacatct   >  1:39703/1‑282`  
                                                                                                                                                                                                                                                                                                                         `GGTCTTTATGTTTAAACTTTAAGATAGGCGTTGACTTGATGGGTCTTTAGGTGTAGGCTTTAGGTGTTGGCTTTAGGATGGACGTTAGGAGGTGACTTTAGGAGGATACTTTAGGAGACTGTAACAGATAGGGACACAGAGAGACACTCAAGGTAACACCCAAAGACAAACCCAAGGGTCATCCCCAACCCTTAGGGAACCCTCGAGGTCAACCGAAGGTTGACTTTAGGTGATTGGCTGGGCTTTAGGTACCCTCTATGGGGGAACTTTAGGTCCGTACctgtctcttatacacatctcc   >  1:27639/1‑280`  
                                                                                                                                                                                                                                                                                                                          `GTCTTTATGTTTAAACTTTAAGATAGGCGTTGACTTGATGGGTCTTTAGGTGTAGGCTTTAGGTGTTGGCTTTAGGATGGACGTTAGGAGGTGACTTTAGGAGGATACTTTAGGAGACTGTAACAGATAGGGACACAGAGAGACACTCAAGGTAACACCCAAAGACAAACCCAAGGGTCATCCCCAACCCTTAGGGAACCCTCAAGGTCAACCGAAGGTTGACTTTAGGTGATTGGCTGGGCTTTAGGTACCCCCTATGGGGGAACTTTAGGTCCGTCCACctgtctcttatacacatctc  >  1:237427/1‑281`  

                                                                                                                                                                                                                                                                                                                                                                                                                                                                                                                                                                                                                                         
`TACTGCATGTTACTTCATGTTGTCAATACCTGTTTTTCGTGCGACTTATCAGGCTGTCTACTTATCCGGTTGACCCCGGTGTCGATTGGGATGGCTATTCGCCGTGTCCCTCTCGATGGCTGTAAGTATCCTATAGGTTAGACTTTAAGTCAATACTCTTTTTGATAAATTTTAAATTAATCTTTTAAGTCTCTTTAAGTTGTCTCTCCTTATAGTGAGTTGTATTAATAAAGACAATGATTAAAGGTTATCACTTTATGGCTAACAACAAGGCGTCTTTAGGTCTGGTCTTTATGTAG‑TGTCTTTAGGTCTGGTCTTTA‑‑‑‑‑‑‑‑‑‑‑‑‑‑‑‑‑‑‑‑‑‑‑‑‑‑‑‑‑‑‑‑‑‑‑‑‑‑‑‑‑‑‑‑‑‑‑‑‑‑‑‑‑‑‑‑‑‑‑‑‑‑‑‑‑‑‑‑‑‑‑‑‑‑‑‑‑‑‑‑‑‑‑‑‑‑‑‑‑‑‑‑‑‑‑‑‑‑‑‑‑‑‑‑‑‑‑‑‑‑‑‑‑‑‑‑‑‑‑‑‑‑‑‑‑‑‑‑‑‑‑‑‑‑‑‑‑‑‑‑‑‑‑‑‑‑‑‑‑‑‑‑‑‑‑‑‑‑‑‑‑‑‑‑‑‑‑‑‑‑‑‑‑‑‑‑‑‑‑‑‑‑‑‑‑‑‑‑‑‑‑‑‑‑‑‑‑‑‑‑‑‑‑‑‑‑‑‑‑‑‑‑‑‑‑‑‑‑‑‑‑‑‑‑‑‑‑‑‑‑‑‑‑‑‑‑‑‑‑‑‑‑‑‑‑‑‑‑‑‑‑‑‑‑‑‑‑‑‑‑‑‑‑‑‑‑‑‑‑‑‑‑‑‑‑‑‑‑‑‑‑‑‑‑‑‑‑‑‑‑‑‑‑‑  <  NC_001604.1/614‑295`  
`‑‑‑‑‑‑‑‑‑‑‑‑‑‑‑‑‑‑‑‑‑‑‑‑‑‑‑‑‑‑‑‑‑‑‑‑‑‑‑‑‑‑‑‑‑‑‑‑‑‑‑‑‑‑‑‑‑‑‑‑‑‑‑‑‑‑‑‑‑‑‑‑‑‑‑‑‑‑‑‑‑‑‑‑‑‑‑‑‑‑‑‑‑‑‑‑‑‑‑‑‑‑‑‑‑‑‑‑‑‑‑‑‑‑‑‑‑‑‑‑‑‑‑‑‑‑‑‑‑‑‑‑‑‑‑‑‑‑‑‑‑‑‑‑‑‑‑‑‑‑‑‑‑‑‑‑‑‑‑‑‑‑‑‑‑‑‑‑‑‑‑‑‑‑‑‑‑‑‑‑‑‑‑‑‑‑‑‑‑‑‑‑‑‑‑‑‑‑‑‑‑‑‑‑‑‑‑‑‑‑‑‑‑‑‑‑‑‑‑‑‑‑‑‑‑‑‑‑‑‑‑‑‑‑‑‑‑‑‑‑‑‑‑‑‑‑‑‑‑‑‑‑‑‑‑‑‑‑‑‑‑‑‑‑‑‑‑‑‑‑‑‑‑‑‑‑‑‑‑‑‑‑‑‑‑‑‑‑‑‑‑‑‑‑‑‑‑‑‑‑‑gtctttaggtctggtctttaTGTTTAAACTTTAAGATAGGCGTTGACTTGATGGGTCTTTAGGTGTAGGCTTTAGGTGTTGGCTTTAGGATGGACGTTAGGAGGTGACTTTAGGAGGATACTTTAGGAGACTGTAACAGATAGGGACACAGAGAGACACTCAAGGTAACACCCAAAGACAAACCCAAGGGTCATCCCCAACCCTTAGGGAACCCTCAAGGTCAACCGAAGGTTGACTTTAGGTGATTGGCTGGGCTTTAGGTACCCCCTATGGGGGAACTTTAGGTCCGTACACTGTGAGA‑‑‑‑‑‑‑‑‑‑‑‑‑  <  NC_001604.1/281‑1`

  

Alignment Legend

Aligned base mismatch/match (shaded by quality score): `ATCG/ATCG` `< 3 ≤` `ATCG/ATCG` `< 11 ≤` `ATCG/ATCG` `< 24 ≤` `ATCG/ATCG` `< 35 ≤` `ATCG/ATCG` `< 38 ≤` `ATCG/ATCG`

Unaligned base: `atcg`    Masked matching base: `atcg`    Alignment gap: `‑`    Deleted base: `‑`

Reads not counted as support for junction

read\_name Not counted due to insufficient overlap past the breakpoint.

read\_name Not counted due to not crossing MOB target site duplication.
